# Indian Polyvalent Antivenom Accelerates Recovery From Venom-Induced Consumption Coagulopathy (VICC) in Sri Lankan Russell’s Viper (*Daboia russelii*) Envenoming

**DOI:** 10.3389/fmed.2022.852651

**Published:** 2022-03-07

**Authors:** Anjana Silva, Fiona E. Scorgie, Lisa F. Lincz, Kalana Maduwage, Sisira Siribaddana, Geoffrey K. Isbister

**Affiliations:** ^1^Department of Parasitology, Faculty of Medicine and Allied Sciences, Rajarata University of Sri Lanka, Saliyapura, Sri Lanka; ^2^Monash Venom Group, Department of Pharmacology, Faculty of Medicine, Nursing and Health Sciences, Monash University, Melbourne, VIC, Australia; ^3^South Asian Clinical Toxicology Research Collaboration (SACTRC), Faculty of Medicine, University of Peradeniya, Peradeniya, Sri Lanka; ^4^Hunter Haematology Research Group, Calvary Mater Newcastle, Newcastle, NSW, Australia; ^5^Department of Biochemistry, Faculty of Medicine, University of Peradeniya, Peradeniya, Sri Lanka; ^6^Department of Medicine, Faculty of Medicine and Allied Sciences, Rajarata University of Sri Lanka, Saliyapura, Sri Lanka; ^7^Clinical Toxicology Research Group, The University of Newcastle, Newcastle, NSW, Australia

**Keywords:** venom induced consumption coagulopathy, Russell’s viper, antivenom, international normalized ratio, fibrinogen

## Abstract

**Background:**

Venom-induced consumption coagulopathy (VICC) is an important clinical consequence of Russell’s viper (*Daboia russelii*) envenoming. There is limited evidence for antivenom effectiveness in resolving VICC. We aimed to compare the recovery of VICC in patients who received and did not receive antivenom following Russell’s viper envenoming.

**Patients and Methods:**

This was a non-randomized observational study comparing patients with VICC from Russell’s viper envenoming given antivenom for systemic envenoming and those not given antivenom. Antivenom administration was decided by the treating physicians. We included 44 patients with confirmed Russell’s viper bites with one or more International Normalized Ratio (INR) value ≥ 1.5 (VICC). We compared five patients who did not receive antivenom with 39 patients who did receive antivenom. The primary outcome was the proportion of patients with an INR < 1.5 by 48 h post-bite.

**Results:**

The antivenom group had higher peak serum venom concentrations [median (IQR) = 272 (96–1,076) ng/mL versus 21 (8–58) ng/mL] and more severe VICC compared to the no antivenom group. Twenty seven of 39 patients (69%) in the antivenom group had an INR < 1.5 at 48 h post-bite compared to none of the five patients (0%) in the no antivenom group (absolute difference: 69%; 95%CI: 13 to 83%; *p* = 0.006; Fisher’s exact test). The fibrinogen recovered in 32 of 39 patients (82%) in the antivenom group compared to one of five patients (20%) in the no antivenom group (absolute difference 62%; 95% CI: 28 to 95%; *p* = 0.001; Fisher’s exact test). Both INR and fibrinogen were significantly improved between 24 and 48 h post-bite in the antivenom group compared to the no antivenom group.

**Conclusion:**

Antivenom accelerated the recovery of VICC in patients with Russell’s viper envenoming, compared to no recovery in a smaller group of patients with milder VICC not receiving antivenom. This supports the efficacy of antivenom in patients with VICC.

## Introduction

Snakebite is a neglected tropical disease that leads to high morbidity and mortality, mainly in South and South-east Asia, sub-Saharan Africa, Latin America and Oceania (1, 2). Venom induced consumption coagulopathy (VICC) is a severe systemic effect of snake envenoming. Bites by most viperidae snakes and Australasian elapid snakes result in VICC in envenomed patients. Potent pro-coagulant toxins in the venoms of these snakes activate the human clotting pathway. This causes rapid consumption of clotting factors, mainly fibrinogen, but also factors V, VIII and X, resulting in varying degrees of consumption coagulopathy, depending on the species of the snake (3, 4). VICC may resolve without sequelae, but it can lead to spontaneous bleeding, and less commonly major hemorrhage (5–8), with intracranial bleeding usually having fatal consequences. The presence of hemorrhagic metalloproteinases in some snake venoms, including Russell’s viper (*Daboia* spp.) and carpet vipers (*Echis* spp.), increases the risk of bleeding because of the additional injury to the vascular endothelium (9).

Antivenoms are polyclonal antibodies of animal origin, raised against snake venoms. They are the only available specific treatment for snake envenoming (4, 10). Despite antivenoms being standard practice for treating VICC, there are no randomized placebo-controlled clinical trials of antivenom for VICC (11). For obvious ethical reasons, previous clinical trials have compared different antivenom treatments without a placebo control group, or are non-randomized studies (4, 11). These studies provide little evidence to support antivenom effectiveness because they compare antivenoms from different manufacturers or different doses of antivenom, and do not compare antivenom with no antivenom (or placebo). In addition, observational studies have polarized opinions on the effectiveness of antivenom therapy for VICC. A study of carpet viper (*Echis* spp.) envenoming showed a recovery of the coagulopathy in patients given antivenom, compared to those who did not receive antivenom, when it was not available (12). In contrast, prospective observational studies of VICC in Australian elapids found that antivenom did not prevent or speed the recovery of the coagulopathy (4, 13), which has also been reported in Papuan taipan bites (14, 15). It is ethically difficult to do clinical trials with placebo arms because antivenom remains the standard of care for coagulopathy in snakebite, so observational studies using convenience or historical controls are required to further explore the role of antivenom in treating VICC from other species of snakes.

Russell’s vipers (*Daboia russelii* and *D. siamensis*) are the most medically important snakes in South and South-East Asia. Both species commonly cause VICC in envenomed patients due to the potent procoagulant toxins in their venoms such as those that activate clotting factors V and X (16). Despite the importance of VICC due to *D. russelii* bites, the effectiveness of antivenom in treating it is unclear (17). Currently, all patients with VICC in Russell’s viper envenoming are treated with antivenom, if VICC is detected. However, in most settings, the presence of VICC is detected based on a positive bedside 20-min whole blood clotting test (WBCT20). Due to the insensitivity of this test to partial VICC (less severe coagulopathy), a proportion of patients with VICC may not be treated with antivenom, because the test is falsely negative (18). This creates a potential comparison group of subjects that are not given antivenom but do develop VICC, which is more often partial VICC. Comparing these patients with the remainder receiving antivenom may help us understand the role of antivenom in treating VICC.

We aimed to investigate the recovery of VICC in confirmed cases of envenoming by *D. russelii* in a Sri Lankan tertiary care center.

## Patients and Methods

We undertook a non-randomized observational study comparing patients with VICC from *D. russelii* envenoming given antivenom to those not given antivenom at a Sri Lankan hospital. These patients were selected from a prospective cohort of adult patients (>16 year) admitted to Teaching Hospital, Anuradhapura, Sri Lanka, following snakebites from August 2013 to October 2014. The cohort study aimed to investigate the clinical effects and epidemiology of snakebite and the design has been previously published (19–23). For this analysis, we included only the cases with authenticated envenoming (see below) by *D. russelii*, whether they received antivenom or not, and had serial samples available for formal coagulation studies.

The prospective cohort study was approved by the Human Research Ethics Committees of the Rajarata University of Sri Lanka (04/09/2013) and Monash University, Australia (CF14/970–2014000404). Written and informed consent for the collection of blood samples and clinical information was obtained from all patients.

All patients aged 16 years or older presenting to Anuradhapura hospital between August 2013 and October 2014 with a snakebite were prospectively recruited to a cohort study. Patients had to have identifiable fang/teeth marks, features of local or systemic envenoming or witness the snake bite to be included. Demographic data is collected on all patients recruited to the snakebite cohort study. Patients had serial clinical examinations and blood sampling on admission and at 1, 4, 8, 12, 24 h after the bite, and daily thereafter. The clinical examination focused on the detection of local and systemic manifestations of envenoming, including bleeding from the gums, hematuria, hematemesis, bite site bleeding and bleeding from intravenous cannula. Neurotoxicity was defined as the presence of signs of neuromuscular paralysis such as ptosis, ophthalmoplegia and facial paralysis. Acute Kidney Injury was defined according to the Kidney Disease: Improving Global Outcomes (KDIGO) criteria (24). All data are collected on a clinical research form while the patient is in hospital by clinical research assistants. Data are then entered into a relational database.

Treating physicians managed all patients, including the decision to administer antivenom, which was based on the presence of systemic envenoming, most often a positive WBCT20 or clinical evidence of systemic envenoming such as neurotoxicity or non-specific systemic symptoms (e.g., abdominal pain). Patients receiving antivenom had an initial dose of 20 vials of Indian polyvalent antivenom raised against *D. russelii, Naja naja, Echis carinatus*, and *Bungarus caeruleus* (VINS bioproducts Telangana, India; Batch numbers, 01AS11118, 1119, 1121, 1123, 3100, 4001, 4025, 4026, 4031). The investigators were not involved in making any treatment decisions. Subsequent doses of antivenom were also decided by the treating physicians based on their assessment of clinical severity of envenoming.

For this non-randomized comparison we only included patients with authenticated *D. russelii* envenoming and VICC, who had citrated plasma samples collected for INR and fibrinogen during the study period. Authentication was based on either the identification of the offending snake specimen by AS (herpetologist) or detection of Russell’s viper venom by enzyme-linked immunosorbent assay (ELISA) (20). We defined VICC as envenomed patients having one or more INR values ≥ 1.5 within the first 24 h post-bite. Complete VICC was defined as an INR > 12 and partial VICC as an INR ≥ 1.5 and < 12. There were 103 patients from the snakebite cohort with definite *D. russelii* envenoming, but only 44 patients had serial citrated plasma samples available for at least 48 h post-bite and an INR ≥ 1.5. Thirty nine patients received antivenom (antivenom group) and five patients did not receive antivenom (no antivenom group). Antivenom was not given in these five patients because they had no features of systemic envenoming and had a negative WBCT20, including on repeat occasions. There were 16 patients who did not receive antivenom, among the 58 patients who were excluded due to the lack of samples up to 48 h. Of the excluded 16 patients who did not receive antivenom, 11 had no samples even up to 24 h.

Citrated samples were stored for serial measurement of prothrombin time (PT or International normalized ratio [INR]) and plasma fibrinogen concentrations. Samples were transported frozen by international courier to the Hunter Haematology Research Group at the Calvary Mater Newcastle, Australia.

### Coagulation Assays

Prothrombin time (PT), international normalized ratio (INR) and fibrinogen concentration were measured in platelet free citrated plasma. All assays were performed using standard coagulometric methods on a Sysmex CS2000i coagulation analyzer (Sysmex Corporation, Kobe, Japan). Briefly, the PT was determined by mixing patient plasma and Innovin reagent (Dade Behring Inc., United States) in a 1:2 ratio, and the time taken for clot formation was measured in seconds. The INR was then automatically derived from the PT according to standard formula. For the fibrinogen assay patient plasma was diluted 1:10 in Owrens Veronal Buffer before being mixed in a 2:1 ratio with Dade Thrombin Reagent (Siemens Healthcare Diagnostic Inc., United States) and time to clot formation was measured in seconds. Fibrinogen concentration was then determined using a standard curve of serially diluted standard human plasma in g/L. The normal range for fibrinogen was defined as 2 to 4 g/L.

### Outcomes

The primary outcome was the proportion of patients whose INR had decreased to <1.5 by 48 h post-bite. The secondary outcomes were: (1) the proportion of patients with a plasma fibrinogen concentration >2 g/L at 48 h post-bite, (2) the change of median INR during the period from 24 to 48 h post-bite, and (3) the change of median fibrinogen for the period from 24 to 48 h post-bite.

### Data Analysis

Continuous variables (fibrinogen, INR, and length of hospital stay) are reported as median values with interquartile ranges (IQR) and ranges. Ninety five percent confidence intervals (CI) are reported for the absolute difference for the outcomes. Dichotomous outcomes were compared using Fisher’s exact test and *p* < 0.05 was regarded as significant. All analyses and graphics were done in GraphPad Prism version 7.02 for Windows, GraphPad Software, San Diego, CA, United States^1^.

## Results

Forty-four patients with VICC were included in the analysis: 39 patients who received antivenom and five patients who did not receive antivenom The two groups had similar demographics, but the antivenom group had more severe envenoming compared to the no antivenom group (Table 1). All patients in the antivenom group received their first dose of 20 vials of antivenom within 6 h of the bite. A further one or more doses of antivenom were administered to 15 (38%) patients. This included 13 patients receiving a total of two doses, one patient each receiving 3 and 4 doses during their hospital stay. None of the patients received fresh frozen plasma or any replacement of clotting factors.

**TABLE 1:** Comparison of the clinico-epidemiological data of the patients with venom-induced consumption coagulopathy following Russell’s viper envenoming recruited for this study: patients who received antivenom (antivenom) and who did not receive antivenom (No antivenom).

	Antivenom (*n* = 39)	No antivenom (*n* = 5)
Age: median (range) in years	40(16−65)	34(17−52)
Sex: males (%)	33 (85)	4 (80)
Bite-to-hospital time in hours: median (IQR)	2(1.5−3.3)	3(1.5−4.4)
Bite-to-antivenom time in hours: median (IQR)	3.8(2.8−4.5)	−
Patients with local features of envenoming (%)	39 (100)	5 (100)
Peak venom concentration: median (IQR) ng/mL	272(96−1,076)	21(8−58)
WBCT20 positive patients (%)	39 (100)	0 (0)
Patients with bleeding manifestations (%)	15 (35)	0 (0)
Patients with complete VICC (%)	20 (51)	0 (0)
Patients with partial VICC (%)	19 (49)	5 (100)
Patients with neurotoxicity (%)	33 (85)	0 (0)
Patients with acute kidney injury (%)	3 (8)	0 (0)
Duration of hospital stay in days: median (IQR)	3(2−4)	2 (2)
Deaths (%)	0 (0)	0 (0)

**FIGURE 1:**
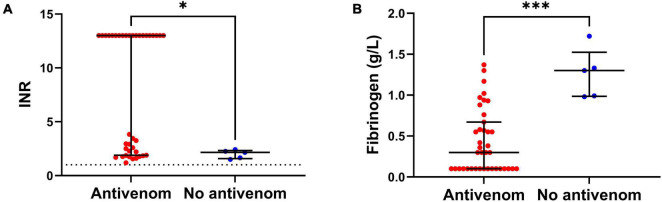
**(A)** Comparison of the peak International Normalized Ratio (INR) of patients from antivenom and no antivenom groups. **(B)** Comparison of the minimum plasma fibrinogen concentrations of patients from antivenom and no antivenom groups. Note, the antivenom group had higher peak INR and lower minimum plasma fibrinogen levels compared to the no antivenom group (*p < 0.05, ^***^p < 0.001, Mann-Whitney test; horizontal bars represent median and interquartile range).

The antivenom group had a higher median peak venom concentration (272 IQR: 96 – 1,076 ng/mL versus 21 IQR: 8 – 58 ng/mL; Table 1). The antivenom group developed more severe VICC with a median highest INR of 13 (IQR, 1.9 – 13) and median lowest fibrinogen concentration of 0.3 g/L (IQR, 0.1 – 0.7 g/L), compared to the no antivenom group which had a median highest INR of 2.2 (IQR 1.6 – 2.3) and a median lowest fibrinogen concentration of 1.3 g/L (IQR, 1.0 – 1.5 g/L) (Figure 1). Twenty (51%) of the antivenom group and none of the non-antivenom group had complete VICC.

Twenty-seven of 39 patients (69%) in the antivenom group had an INR < 1.5 at 48 h post-bite compared to none of the patients (0%) in the no antivenom group (absolute difference: 69%; 95%CI: 13 to 83%; *p* = 0.006). The fibrinogen recovered in 32 of 39 patients (82%) in the antivenom group compared to one of the five patients (20%) in the no antivenom group (absolute difference 62%; 95% CI: 28 to 96%; *p* = 0.001).

Comparing only patients with partial VICC, 14 of 19 (74%) patients in the antivenom group had an INR < 1.5 at 48 h post-bite, compared to none of the patients (0%) in the no antivenom group (absolute difference: 74%; 95% CI: 14 to 90%; *p* = 0.006). The fibrinogen recovered in 15 of 19 (79%) patients in the antivenom group compared to one of the five patients (20%) in the no antivenom group (absolute difference 59%; 95% CI: 3 to 83%; *p* = 0.028).

**FIGURE 2:**
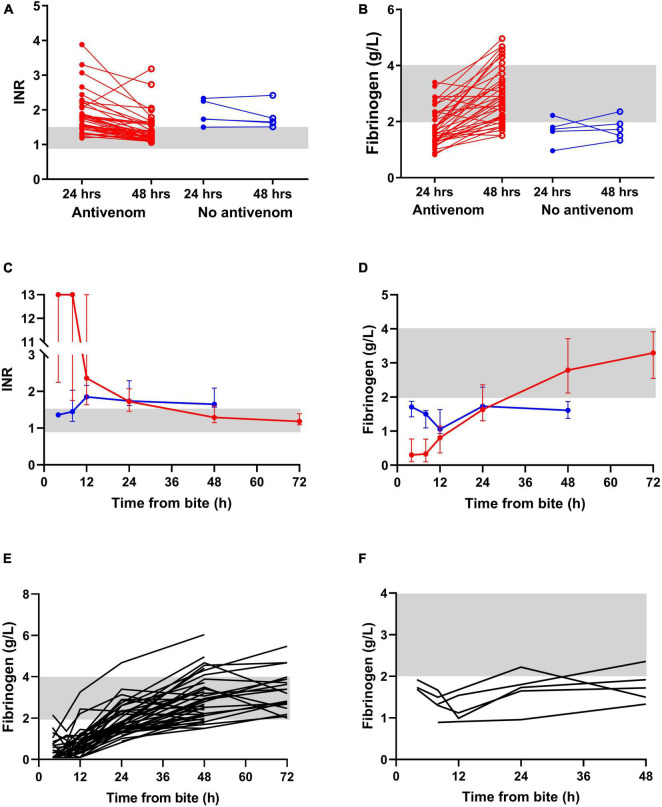
Change of the INR and fibrinogen concentrations of antivenom (red symbols) and no antivenom (blue symbols) groups: change of INR **(A)** and fibrinogen **(B)** between 24 and 48 h from the bite in antivenom and no antivenom groups (note both INR and fibrinogen significantly improve between 24 and 48 h period in the antivenom group as opposed to no improvement in no antivenom group, *p* ≤ 0.0001, paired *t* test); **(C)** Time-related change of the median and Interquartile range of INR **(C)** and fibrinogen **(D)** of antivenom and no antivenom groups; noodle plots showing the time-related change of plasma fibrinogen concentrations of all patients of antivenom **(E)** and no antivenom **(F)** groups. The shaded areas represent the normal range.

International normalized ratio was significantly reduced (change in INR) between 24 h and 48 h post-bite in the antivenom group compared to the no antivenom group (median change in INR of 0.4 versus 0.1; *p* = 0.033 Mann-Whitney; Figures 2A,C). Similarly, fibrinogen concentrations were significantly improved (increase in fibrinogen) between 24 and 48 h post-bite in antivenom group compared to no improvement in the no antivenom group (median change in fibrinogen of 1.1 versus 0.2; *p* = 0.008 Mann-Whitney; Figure 2B). Serial fibrinogen concentrations increased over the admission for those treated with antivenom compared to those not treated (Figures 2D–F).

## Discussion

We found that VICC in Russell’s viper envenoming recovers within 48 h in patients who received antivenom within 12 h of the bite, compared to those who did not receive antivenom not recovering. Administration of antivenom resulted in fibrinogen concentrations increasing and the INR decreasing into the normal range. Although the patients that did not receive antivenom had milder or partial VICC, the coagulopathy persisted for more than 48 h post-bite. In contrast, the antivenom group initially had higher venom concentrations, more severe coagulopathy, but there was a rapid recovery in both fibrinogen and INR.

There have been several previous observational studies on the Russell’s viper envenoming in Sri Lanka, that concluded ineffectiveness or did not conclude effectiveness of the Indian polyvalent antivenom in treating VICC in Russell’s viper envenoming in Sri Lanka (25–27). Unlike the previous studies, we included a comparison group of patients not treated with antivenom and had serial measurements of INR and fibrinogen that demonstrated a faster recovery of VICC in the antivenom group. The Russell’s viper venom contains potent pro-coagulant toxins which are activators of clotting factors V and X, leading to rapid consumption of clotting factors resulting in hypofibrinogenaemia (17). It appears that in the absence of antivenom, there is a persistence of active toxins in the circulation and ongoing consumption of clotting factors in patients with partial VICC as they are newly synthesized by the liver. In patients receiving antivenom, the antibodies presumably bind with the procoagulant toxins in the circulation, which facilitates their neutralization and elimination. This allows replacement of clotting factors by the liver and recovery of clotting function (4).

These findings again demonstrate important differences between VICC caused by different snakes, and whether antivenom is beneficial for a particular group of snakes. Previous studies have demonstrated that antivenom has little effect on the recovery of VICC in Australasian elapids (13–15), but others have shown it is effective in treating VICC caused by *Echis* spp. (12). The latter study on VICC in carpet viper (*Echis ocellatus*) envenoming included an untreated group who had persistent severe coagulopathy for 8 to 10 days, compared to a group receiving antivenom, who had a rapid recovery within 24 to 48 h (12). The untreated group had similarly severe VICC compared to the antivenom group in this study and had a prolonged observation period, making the differences in the timing of recovery from VICC more pronounced. In this study, we have found that similar to *Echis*, another true viper (Viperinae), VICC resulting from *D. russelii* takes longer to recover in patients not treated with antivenom. The effectiveness of antivenom cannot be generalized for a particular toxidrome (i.e., VICC), and studies are required for each group of snakes and their respective antivenoms.

There are several limitations to the study, the major one being the small number of patients not receiving antivenom and that VICC in these patients was mild, compared to those treated with antivenom. However, we would expect a rapid recovery in these patients with mild VICC who did not receive antivenom, but this was not the case with little recovery over 48 h (Figure 2). In contrast, the more severe VICC seen in the patients given antivenom recovered rapidly, with over half having normalized clotting function within 48 h. In addition, a sensitivity analysis, which only included patients with partial VICC in each group, we found a significant difference between those treated with antivenom and those not treated.

Serial INR and fibrinogen concentrations are required for at least 48 h post-bite to assess restoration of blood coagulability. Many of the patients not receiving antivenom are discharged earlier, usually after only a 24 h observation period. This was the reason for the low number of patients in the no antivenom group in this study, but also means that there are patients being discharged from hospital with persistent mild to moderate coagulopathy. This meant that recovery back into the normal range was not observed for many of the patients not receiving antivenom.

Another possible limitation of the study was that the patients not treated with antivenom had only mild or partial VICC, and it could be argued that they do not require treatment. However, there is increasing evidence that thrombotic microangiopathy and acute kidney injury can occur following partial VICC with only a mild coagulopathy, often with a negative WBCT. This is seen in both Australia, with brown snake bites (6), as well as with viper bites in South Asia. A study of acute kidney injury in Russell’s viper bites found that 5 of 24 patients with AKIN Stage 1 and 5 of 13 patients with AKIN Stage 2 had a normal WBCT, similar to the group in our study (28). Similarly, in hump-nosed viper bites, the majority of patients have a normal WBCT and minor systemic symptoms, but a small proportion develop AKI, not always with an abnormal WBCT (29–32). Although many of these patients with mild VICC may not develop complications, previous studies demonstrate that there is a small but important group of patients with mild VICC who go on to develop AKI, often requiring dialysis and rarely being fatal (31, 32). In addition, Sri Lankan Russell’s viper venom contains metalloproteinases that induce hemorrhage as evident from functional venomic studies (33, 34), hence there is a risk of spontaneous bleeding despite having partial VICC. Therefore, any patient with evidence of VICC should be considered to be systemically envenomed and therefore require antivenom.

This study again shows that due to the lesser sensitivity of WBCT20 for milder VICC in particular (35), some patients who might require antivenom therapy are left untreated and discharged early. Studies have shown that milder VICC (INR 1.5-3) is not clinically insignificant, and antivenom may prevent complications such as AKI and thrombotic microangiopathy.

## Conclusion

Venom-induced consumption coagulopathy that develops in patients with Russell’s viper envenoming recovers in the first 48 h if treated with antivenom. Patients that had less severe or partial VICC that did not receive antivenom, still had persistent coagulopathy at 48 h. Our study provides evidence for the efficacy of Indian Polyvalent antivenom in quickening the recovery of VICC caused by Russell’s viper envenoming. In the absence of evidence originating from randomized-controlled trials (11), the evidence generated from this study is useful in better determining the role of antivenom in the treatment of VICC.

## Data Availability Statement

The raw data supporting the conclusions of this article will be made available by the authors, without undue reservation.

## Ethics Statement

The studies involving human participants were reviewed and approved by the Human Research Ethics Committees of the Rajarata University of Sri Lanka (04/09/2013) and Monash University, Australia (CF14/970–2014000404). The patients/participants provided their written informed consent to participate in this study.

## Author Contributions

AS and GI: conceptualization, formal analysis, and writing – original draft preparation. AS, FS, LL, KM, SS, and GI: methodology. AS, FS, and KM: investigation. GI and LL: resources. AS and SS: data curation. FS, LL, KM, and SS: writing, review, and editing. GI and SS: supervision. GI: funding acquisition. All authors have read and agreed to the published version of the manuscript.

## Conflict of Interest

The authors declare that the research was conducted in the absence of any commercial or financial relationships that could be construed as a potential conflict of interest.

## Publisher’s Note

All claims expressed in this article are solely those of the authors and do not necessarily represent those of their affiliated organizations, or those of the publisher, the editors and the reviewers. Any product that may be evaluated in this article, or claim that may be made by its manufacturer, is not guaranteed or endorsed by the publisher.

## Footnotes

1
www.graphpad.com
